# Exhaled nitric oxide in diagnosis and management of respiratory diseases

**DOI:** 10.4103/1817-1737.56009

**Published:** 2009

**Authors:** Abdullah A. Abba

**Affiliations:** *Department of Medicine, College of Medicine and King Khalid University Hospital, Riyadh, Saudi Arabia*

**Keywords:** Diseases, exhaled nitric oxide, measurement, respiratory

## Abstract

The analysis of biomarkers in exhaled breath constituents has recently become of great interest in the diagnosis, treatment and monitoring of many respiratory conditions. Of particular interest is the measurement of fractional exhaled nitric oxide (FENO) in breath. Its measurement is noninvasive, easy and reproducible. The technique has recently been standardized by both American Thoracic Society and European Respiratory Society. The availability of cheap, portable and reliable equipment has made the assay possible in clinics by general physicians and, in the near future, at home by patients. The concentration of exhaled nitric oxide is markedly elevated in bronchial asthma and is positively related to the degree of esinophilic inflammation. Its measurement can be used in the diagnosis of bronchial asthma and titration of dose of steroids as well as to identify steroid responsive patients in chronic obstructive pulmonary disease. In primary ciliary dyskinesia, nasal NO is diagnostically low and of considerable value in diagnosis. Among lung transplant recipients, FENO can be of great value in the early detection of infection, bronchioloitis obliterans syndrome and rejection. This review discusses the biology, factors affecting measurement, and clinical application of FENO in the diagnosis and management of respiratory diseases.

The powerful substance found in the walls of veins and arteries and originally named endothelium-derived relaxing factor was shown in 1987 to possess the same biological and chemical properties as nitric oxide (NO), a molecule with a simple atomic structure.[1,2] The demonstration of the presence of NO in exhaled air by Gustafsson *et al.* in 1991[3] led to numerous publications on the subject and the molecule was subsequently named “molecule of the year” in 1992 by the journal Science. [4] There is a body of evidence showing that the fraction of exhaled nitric oxide (FENO) reflects the degree of inflammation, particularly esinophilic inflammation, in the airways.[5–8]

Chemiluminescence and electrochemical analyzers can measure NO. Cheaper and more portable NO analyzers are now readily available and increasingly being used for the assay of FENO. American Thoracic Society and European Respiratory Society have jointly published recommendations for the procedure of measurement of both exhaled and nasal NO to allow standardization of measurement and for the comparison of results from various centers.[9] In bronchial asthma for example the adequacy of control of inflammation is often at variance with symptoms, physical signs, airflow limitation(as measured by spirometry) and bronchial hyper responsiveness. The availability of a simple, noninvasive test of inflammation has therefore attracted tremendous interest in pulmonology.

This paper reviews the biology of NO, methodology of assay and factors affecting the measured values. We also discuss the clinical application of FENO in respiratory diseases.

## Background

Nitric oxide is synthesized by the conversion of the amino acid l-arginine to l-citrulline and NO by the enzyme NO synthase (NOS).[10] There are three isoforms of NOS. Two, neuronal isoform (nNOS) and endothelial (eNOS), are constitutive and calcium dependant. Both are found in the airway epithelium where they produce picomolar concentrations of NO. The third is inducible (iNOS) and calcium-independent. This is expressed *in vivo* in the bronchial epithelial cells in both healthy and asthmatic individuals and its activity increases during certain inflammatory processes. It is also expressed *in vitro* following stimulation by cytokines, endotoxins and lipospolysaccharides. Inducible NOS produces nanomolar concentrations of NO which remains stable in the gaseous phase and can be assayed.[11]

**Figure 1:**
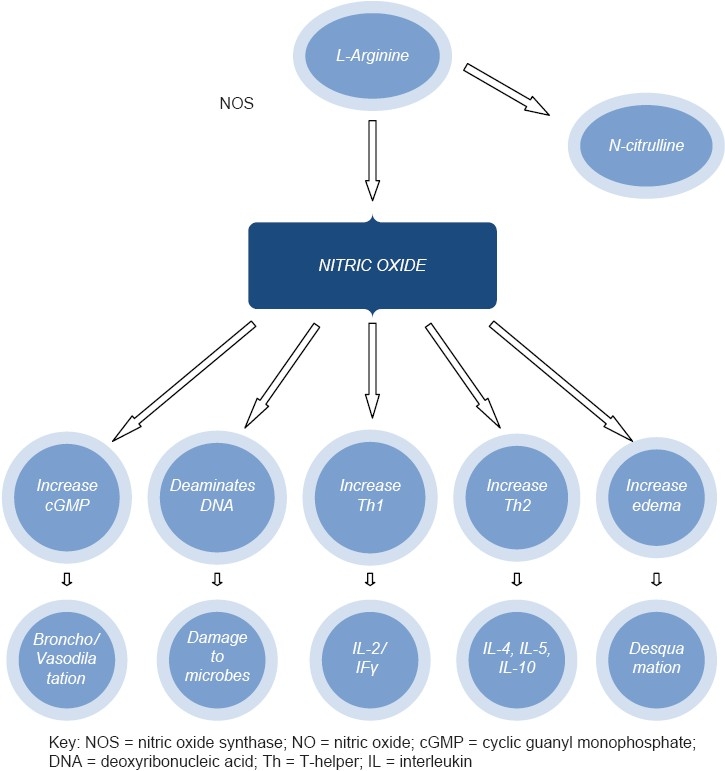
Synthesis and functions of nitric oxide

NO has numerous functions in the airways. It has a weak bronchodilator effect through the stimulation of the enzyme guanylate cyclase which increases the production of cyclic guanine monophospahte (cGMP) which in turn leads to relaxation of the bronchial smooth muscle. Through the same mechanism it leads to vasodilation in the pulmonary vessels. NO also functions as a neurotransmitter of the noradrenergic noncholinergic (NANC) system, the only neural bronchodilator mechanism. By its ability to damage intracellular pathogens, NO is associated with increased host resistance. It deaminates deoxyribonucleic acid (DNA) and damages lipids in the membranes of the microorganisms. Furthermore, increased local concentration of NO tends to increase T-helper 2 (Th2) cells (secrete interleukins IL-4, IL-5 and IL-10 that promote the production of immunoglobulin E (IgE) and adherence and accumulation of esinophils and inhibits the differentiation of CD4 T-helper cells into Th1 (producers of IL-2 and interferon gamma). This is the pattern seen in both atopic and non atopic asthmatic patients and is effectively inhibited by corticosteroids.[12,13] Lastly, NO tends to increase edema and plasma exudation and cause denudation and desquamation of the epithelial lining [Figure 1].

## Factors Affecting FENO Measurement

NO in the airway is measured by its reaction with ozone which is detected by chemiluminescence. The technical aspects of measurement of FENO had been published by the European Respiratory Society (ERS) and American Thoracic Society (ATS) in the 1990s; further updated and standardized in 2005[9,14,15] and beyond the scope of this review. Suffice it to say that FENO can be measured online during slow expiration or offline from samples of exhaled air collected in bags. The former method is applicable to patients who are able to cooperate, the latter particularly useful in children under four years. The standard expiratory flow rate of 50mL/s is set for online measurement as figures vary depending on the flow rate: The rate of NO output is greater at higher flow rates analogous to respiratory heat loss. Due to the flow dependency, standard techniques emphasize the need for using a constant expiratory flow rate. Exhalation should be against closed palate to avoid contamination by nasal NO.

A number of demographic, anthropometric and biological factors cause variation in the levels of FENO. There are, however, conflicting reports on the significance of the various factors in the measurement of FENO complicating the definition of normal values.

1. **Age*:*** A consistent positive correlation between age and values of FENO[16,17] is observed in children who have an age, body size relationship. This reflects the increasing surface area of the airways with increasing age. In adults, however, the data is conflicting. The largest study so far on the factors affecting FENO in adults found age independently and positively associated with FENO. The oldest group (over 64 years) had a 40% higher FENO value as compared to the youngest group (35- 44 years)[18]. However, Travers *et al.* and Olivieri *et al.* did not observe any significant relationship between age and the values of FENO in adults.[19,20]
2. **Gender*:*** Earlier studies have yielded conflicting results on the effect of gender on FENO. While some studies have found as much as 1.26 times higher figures in males than females,[19,20] others have found no difference if the other anthropometric factors are taken into consideration.[18,21] A more recent study by Taylor *et al.* revealed that the effect of sex is both statistically and clinically significant with FENO levels approximately 25% less in females after controlling for all other significant factors affecting FENO.[22] It is not clear why there is a difference between the sexes but factors related to hormone production seem to be unlikely as demonstrated by Morris *et al.*[23] Differences in the airway caliber and surface area may be responsible for the observed variation. The same flow rate in airways of varying calibers may variably dilute NO moving by gaseous diffusion into smaller lumen in females, leading to lower NO concentration[17,24] Buchvald *et al.* found that FENO levels were similar between boys and girls of the same age but there was a significant and positive correlation between levels and age.[16] Since airway surface area increases with age in both sexes, this may suggest that the low FENO levels in women may simply be an artifact due to the use of a constant exhaled flow rate rather than a real reduction in NO airway production. Clearly, more data on the effect of sex is needed for proper interpretation of routine FENO measurement.
3. **Anthropometric factors and race:** Olin *et al.* noted that FENO was clearly positively correlated with height in both males and female adults.[18] This is probably because of the height-dependant increase in the total airway mucosal surface area that produces NO.[25] In a study of 657 children, Kovesi *et al.* found FENO values to be significantly positively correlated with height (*P* = 0.023), age (*P* = 0.007) and race[26] but not with body mass index (BMI). Also in a study of 114 children, Malberg *et al.* found FENO significantly associated with age, height, weight and BMI (*P* < 0.0001); height being the strongest independent predictor.[27] Other studies, however, did not find any correlation between anthropometric values and levels of FENO in both children and adults.[17,20] In a study of normal adults, we observed a negative correlation between the BMI and FENO levels (unpublished data). Nevertheless, the joint ATS/ERS recommendations require documentation of these variables.[9] Racial variation has been noted by some workers. Buchvald and Wong observed that non White and Chinese subjects had higher values of FENO than Whites.[16,28] Kovesi observed that Asian children had higher values than Whites and that Blacks had an intermediate value.[26] There is no identifiable genetic explanation for these differences.[29]
4. **Smoking and dietary factors:** Smoking has been shown to reduce the level of FENO both acutely and on long term basis.[30,31] Conversely, cessation of smoking has been shown to increase the level of FENO.[32] It is believed that the effect is through down regulation of both endothelial NO synthase (eNOS) and inducible NO synthase (iNOS).[33] A record of smoking history is therefore necessary for the interpretation of results and measurement is advised at least an hour after smoking. An increase in FENO has been shown after ingestion of nitrate rich food[34] such as lettuce and radishes, drinking of water and ingestion of caffeine.[35,36] A positive correlation has also been noted between the levels of FENO and the dietary consumption of fats and antioxidants.[37] Alcohol has been shown to decrease the level of FENO.[38] It is therefore prudent to refrain from eating and drinking for at least one hour before measurement.
5. **Medication:** A number of medications affect levels of FENO. Inhaled and oral corticosteroids as well as leukotriene-axis modifiers such montelukast reduce the levels in subjects with asthma.[39–41] L-arginine increases the levels of FENO as do NO donor drugs such as nitrates.[42–44] Other drugs affect the apparent levels of FENO by other mechanisms such as changes in the airway caliber. Bronchodilators like B-agonists have been shown to increase[45] while bronchoconstriction leads to reduction in FENO[46] levels. These changes are believed to be due to mechanical effect on airway NO production.
6. **Circadian rhythm and seasonal variation:** It is not certain whether measurement needs to be standardized for time of day. While most studies have indicated that there is no circadian or day-to-day variation,[47–49] at least one study reported a circadian pattern.[50] Seasonal variations in values of FENO due to fluctuation in exposure to allergens have also been reported in patients with allergic asthma and allergic seasonal rhinitis.[51] It is therefore prudent to note the season and time of measurement and if possible measure the values at a particular time of the day.
7. **Other factors:** Levels of FENO tend to decrease after spirometry,[45] sputum induction,[9] bronchoprovocation study[52] and within 30 minutes of exercise.[53] Adequate time should therefore be allowed before measurement if any of these circumstances prevail.

## Reference Values of FENO

The establishment of reference values for a population is difficult because of the numerous confounding factors as discussed above. Until now only a few studies examined the values of FENO in more than 50 adults using current standard methods as recommended by ATS/ERS.[16,19–21,54] In the largest study so far on reference values involving 3,376 adults, Olin *et al.* defined a value of 24.0 to 54.0 ppb depending on age and height.

The reference equation for healthy nonsmoking adults was defined as: Ln (FENO) = 0.057 + 0.013 × height (in centimeters) + 0.0088 × age (in years).

For non atopic subjects alone, the equation was Ln (FENO) = −0.0026 + 0.013 × height (in centimeters) + 0.010 × age (in years).[21]

Table 1 gives a summary of the reference values obtained among adults and children. We observed a figure of between 7.66 and 46.6 ppb among nonsmoking, nonatopic adult male Saudi subjects (unpublished data). Before interpreting the values of measured FENO, the clinician has to be aware of the methods employed in the measurement and any confounding factors. At the moment there are no set cutoff limits but where available local reference figures should be used for the interpretation and clinical application. It is probably more practical to take into consideration individual readings in monitoring of disease state and response to therapy.

**Table 1:** Reference values of fraction of exhaled nitric oxide

Authors/year of publication[ref]	Number/type of subjects	Reference range (ppb)	Comments
Olin *et al.*, 2007[21]	3,376; Adult neversmokers	24.0-54.0	Equation: Ln (FENO) = 0.057 + 0.013 × height (cm) + 0.0088 × age (years)
Travers *et al.,* 2007[19]	3,500; Adults	7.8-41.1	Geometric mean 17.9 Ppb
Olivieri *et al.,* 2006[20]	204, Adult non-smokers	2.6-28.8 Males 1.6-21.5 Females	-
Daniel *et al.,* 2007[54]	121, children 2 to 7 years	1.2-8.2 Online,1.3-7.1 Off line,	Geometric mean 3.9 Ppb online, 3.0 Ppb off line
Buchvald *et al.,* 2005[16]	405, children 4 to 17 years	15-25, Geometric mean 9.7	Significant increase with age
Franklin *et al.,* 1999[17]	157, healthy children	Geometric means: 7.22B ppb no spt, 1 spt 10.9, 2 Or more spt 20.1	Different mouth pressures used than current standard of 50 ml/second

Ref = reference number in text, ppb = parts per billion, SPT = skin prick test

## Clinical Application of FENO

The availability of cheap, portable and reliable equipment for the measurement of FENO has led to widespread use of FENO as a noninvasive diagnostic and management tool particularly in bronchial asthma. A number of factors and disease states affect the measured values of FENO. The levels could be increased, decreased or variably affected [Table 2]. Of these conditions, we shall discuss clinical application of FENO in the diagnosis and management of: Asthma, atopy, COPD, bronchiectasis, cystic fibrosis, primary ciliary dyskinesia, interstitial lung disease, pulmonary hypertension and lung transplantation.

**Table 2:** Diseases that modify fraction of exhaled nitric oxide

Increase	Decrease	Variable
Asthma ± atopy	Cystic fibrosis	Bronchiectasis
Allergic rhinitis	Primary biliary dyskinesia	Stable COPD
COPD ± exacerbation	Pulmonary	Fibrosing
	hypertension	alveolitis
Bronchiectasis	Pneumonia	Systemic
		sclerosis
Primary lung cancer	GERD	Sarcoidosis
Lung transplant (acute rejection)	Laryngeal tracheomalacia	
Rhinoviral infection/urti	HIV infection	
Pulmonary	Diffuse alveolar	
tuberculosis	hemorrhage	
Liver cirrhosis/hepatopulmonary		
syndrome		
Chronic inflammatory		
bowel disease		
SLE		

COPD = Chronic obstructive pulmonary disease

URTI = Upper respiratory tract infection

SLE: Systemic lupus erythrematosus

GERD = Gastroeosophageal reflux disease

### Bronchial asthma

In subjects with asthma, the levels of FENO have been shown to have excellent correlation with esinophilic airway inflammation as represented by blood, sputum, bronchoalveolar lavage (BAL) and mucosal esinophilia.[55–58] It is considered a valid and reproducible noninvasive marker with a high discriminatory capacity and can be used with more than 90% specificity for the diagnosis of asthma in both adults and children.[59,60] Malberg *et al.* observed that high FENO values (greater than or equal to 3SD) correlated with clinical asthma (odds ratio 16.3; 95% CI5.4 minus 49.7; *P* < 0.0001). Furthermore, in a comparison of spirometry, forced oscillometry, bronchodilation test, and induced sputum, they found that the diagnostic sensitivity of forced spirometry was lower (47%) than that of either FENO (88%) or induced sputum (86%).The specificity of FENO was 92%.[61] The elevation of FENO in patients with other atopic conditions such as hay fever and eczema and the normal values seen in children with virus-induced asthma reduce the diagnostic utility of the test in this group.[62]

In the area of monitoring asthma, FENO correlates with symptom frequency and bronchodilator use but not with FEV1.[63] In patients with subclinical airway inflammation in whom the FENO values are elevated, early anti inflammatory treatment may prevent subsequent remodeling and progression of asthma.[57] FENO values rapidly decrease with the use of inhaled corticosteroids and anti leukotriene therapy but not with neodocromil or theophylline.[64] It can therefore be used to predict the response to, and titrate the dose of, steroids. Furthermore, it can be used as a marker of loss of control and identify lack of adherence to anti-inflammatory medications.

A recent meta analysis of the value of tailoring the dose of inhaled corticosteroids in the management of asthma based on the FENO levels in comparison to the traditional methods of using symptoms and spirometry found that the differences are only modest. Four studies (two adult and two pediatric) were included; these studies differed in a variety of ways including definition of asthma exacerbations, FENO cutoff levels and duration of study. Of 356 participants randomized, 324 completed the trials. In the meta analysis, there was no difference between groups for the primary outcome of asthma exacerbations or for other outcomes (clinical symptoms, FENO level and spirometry). In a post hoc analysis in adults, the group with treatment based on FENO had a significant reduction in mean final daily dose inhaled corticosteroid compared to the group whose treatment was based on symptoms (WMD −282.46, 95% CI −422.08 to −42.84). There were no differences in the adult or pediatric studies between the groups in the overall daily ICS dose.[65] The measurement of FENO may therefore be of value in the diagnosis and monitoring of treatment of bronchial asthma.

### Atopy

Atopy often predates bronchial hyper responsiveness and asthma and there is evidence that it is associated with airway inflammation as manifested by elevated FENO values.[66] Among adults, asthmatic patients with atopy have significantly higher levels of FENO than nonatopic asthmatics.[67] Similarly, in children, the level of FENO is higher in atopic subjects irrespective of whether they have asthma or not[68] - although atopic-asthmatic children tend to have higher levels of FENO than atopic individuals.[69] The presence of allergic rhinitis also tends to significantly raise the levels of FENO.[70] These factors therefore need to be taken into account in the interpretation of FENO levels. They also make the measurement of FENO a less useful screening tool for identifying individuals with asthma in the community.

### Chronic obstructive pulmonary disease

Data on FENO in chronic obstructive pulmonary disease (COPD) is conflicting and therefore the measurement is less useful in clinical practice. Some studies reported an increase in values in patients with stable COPD,[71–73] while others have shown reduced or unchanged values.[74–76] The levels of exhaled NO have been shown to be inversely related with FEV1, diffusion capacity of carbon monoxide (DLCO) and oxygen saturation (SaO2) and positively with the residual lung volume/total lung capacity ratio.[72] The progression of COPD from GOLD stage 0 to 4 is most strongly associated with thickening of the wall of the small airways by a repair or remodeling process.[77] Brindicci *et al.* found a significant correlation between alveolar NO (CalvNO) and both FEV1 and FEV1/FVC ratio.[73] This indicates that alveolar concentration of NO (CalvNO) in COPD patients may reflect peripheral inflammation and remodeling thus making its measurement a potential powerful tool in diagnosing early stages of peripheral inflammation. Ziora *et al.* on the other hand did not demonstrate any correlation between FENO and other markers of inflammation in induced sputum such as nitrogen oxides (nitrite or nitrate) and cytokines (IL-8, TNF-α, TGF-β1, GM-CSF, eotaxin) except for a negative correlation with the cytokine GM-CSF (r = −0.38, *P* = 0.02) in patients with all stages of COPD.[78]

Kunisaki *et al.* have recently shown that in ex-smokers with severe COPD, FENO, may be more closely associated with FEV1 responses to four weeks of ICS than are standard markers of systemic inflammation namely serum c-reactive protein (CRP), IL-6, and IL-8.[79] FENO may have a role in the monitoring of anti-inflammatory therapy of COPD and can be used to identify patients who may respond to steroids. Clearly, further studies are needed in this area.

### Bronchiectasis, primary ciliary dyskinesia, cystic fibrosis

While some studies have shown an increase in FENO in patients with stable bronchiectasis and a relationship with the extent of disease according a CT score, others have shown similar or lower values compared to normal subjects.[80–83] Lower values of NO have been recorded in airway NO as opposed to alveolar NO[83] and in association with *Pseudomonas aueroginosa* infection. [82,84] However, studies have consistently revealed low levels of exhaled NO in primary ciliary dyskinesia (PCD); the low levels being attributed principally to the reduced bronchial iNOS activity rather than the alveolar eNOS.[85,86] In another study, low values in both eNO and nasal NO readings identified PCD patients from other bronchiectatic patients with a specificity of 98% and positive predictive value of 92% making the simultaneous measurement of exhaled NO and nasal NO a useful screening tool for PCD.[87] In cystic fibrosis, in spite of the intense inflammation in the airways, eNO levels have been found to be the same or lower than in controls.[88,89] This may be due to alteration in NO diffusion through thick mucus, removal of NO by reaction with reactive oxygen species in the inflamed environment and failure of up regulation of epithelial iNOS in chronic suppurative conditions. The dramatic reduction of iNOS expression in airway and nasal epithelium in CF patients may hinder an important first-line defense mechanism and increase the susceptibility of the airways to bacterial infections. The low nasal NO concentrations are probably due to impaired flow from the para nasal sinuses.

Nasal NO is of considerable value in the diagnosis of PCD, but FENO appears to be of limited value in the diagnosis of PCD and cystic fibrosis.

### Interstitial lung disease

Measurement of FENO will be of value in identifying patients with active inflammation and/or pulmonary hypertension who may be amenable to treatment. In a comparative study of patients with cryptogenic fibrosing alveolitis and systemic sclerosis (SSc) with lung fibrosis, FENO was elevated in both groups and correlated with the presence of active bronchoalveolar lavage (BAL) fluid compared to normal nonsmoking subjects or patients on corticosteroids.[90] The level of FENO is increased in patients with SSc and this is believed to originate from the alveolar concentration (Calv) because of the reduced diffusion capacity of NO rather than the airway concentration. The increase is inversely related to the total lung capacity and DLCO and directly related to the computerized tomogram (CT) scan fibrosis score. Calv may therefore be used to non-invasively assess extent of interstitial lung disease in SSc.[91,92] Similarly, in asbestosis and asbestos pleural plaques, Calv has been shown to be increased and may be used to measure inflammation.[93,94]

In patients with pulmonary sarcoidosis, the largest study to date involving 52 patients, showed that FENO is not increased in active disease and that there is no relationship of FENO to the extent of lung fibrosis as measured by CT scan.[95] These findings support earlier smaller studies.[96,97] One other study showed an increase in FENO but no relationship to severity of disease.[98] and another small study showed an increase in FENO in newly diagnosed cases of pulmonary sarcoidosis which fell with treatment.[99]

Measuring the Calv FENO can be of value in the diagnosis and monitoring of lung fibrosis in SSc.

### Pulmonary hypertension

Pulmonary hypertension is believed to be a consequence of reduced production of vasodilator substances such as nitric oxide and FENO measured over time can be used as a non- invasive marker of severity of pulmonary arterial hypertension (PAH) and response to therapy. There is evidence that there is reduced airway wall concentration of NO in patients with pulmonary hypertension.[100,101] Endogenous NO is inversely related to pulmonary artery pressure in PAH and successful therapy of PAH is associated with increase in NO. In patients with COPD, the levels of FENO in patients with PAH were found to be significantly lower than those without PAH or controls. Conversely, the levels of endothelin-1 (ET-1) was higher in patients with PAH than those without or controls suggesting that pulmonary hypertension was a consequence of an imbalance in the output of the two agents by pulmonary epithelium and endothelium.[102] The levels of FENO have been found to correlate to the degree of PAH as assessed by Echo-Doppler studies.[103]

### Lung transplantation

In patients with lung transplantation (LTr), it is important to detect infection, bronchiolitis obliterans syndrome (BOS) and rejection early. This is because early institution of treatment impacts on the outcome. Ltr with pulmonary or upper airway infections had significantly higher FENO than controls and the levels fell with treatment. The sensitivity and specificity of FENO measurement in detecting pulmonary infections were 57 and 96% respectively making the measurement of FENO of limited value as a screening tool for infections because of it low sensitivity.[104] BOS is the greatest threat to long-term outcome after LTr. Early and late acute rejection, cytomegalovirus infection and airway ischemia are major factors for the development of BOS. Its early detection and prompt treatment is necessary to improve survival. High FENO has been shown to be of high negative predictive value but low specificity and positive predictive value in the detection of BOS stages 0-p and 1.[105] Another study has shown the measurement to be accurate in the detection of early BOS.[106] Serial measurement of FENO has been shown to be of value in the prediction of the development of BOS and its deterioration.[107,108] FENO has also been shown to correlate with the degree of acute lung allograft rejection as distinct from infection or BOS.[109,110]

The measurement of FENO is therefore of great potential benefiting the management of patients with LTr.

## Conclusion

The measurement of FENO is dependent on many confounding factors, however the availability of cheap and reliable equipment will make it more commonly used and refined. It is of value in the diagnosis of asthma, steroid responsive COPD, PCD, cystic fibrosis and interstitial lung disease and systemic sclerosis. Furthermore, it has proved useful in diagnosing and monitoring complications in lung transplant patients. Further studies are needed to identify its role in other conditions and the cost-effectiveness in routine clinical practice.
